# Laboratory Methods

**Published:** 1907-10-05

**Authors:** 


### Points in Pathology.

### LABORATORY METHODS.

### Colour Preservation of Macroscopic Specimens.

It often happens that one would like to preserve a specimen obtained at autopsy, or by surgical removal, in such a way as to keep its colour. The specimens upon the shelves of our museums—at any rate the majority of the specimens there—look very different in their bottles to what similar specimens are like in the post-mortem room; the main difference between the two is that the preserved specimen has lost all the varied colouring that the fresh organ possesses. The hospitals are altering this now; specimens are being "kept for colour" to a greater extent every day.

It is not only in hospitals, however, that specimens are kept; many a doctor preserves the more interesting things that he comes across in practice, either for his own personal interest, or else to show them to others at meetings of societies, and so forth. These specimens are the more interesting the more nearly they approach to their original shape and appearance. The difficulty is that, unlike a hospital, a doctor in private practice is not preserving specimens every day, and when the occasion suddenly arises when he would like to, he is at a loss what to do beyond putting the tissue direct into alcohol, formaline, or some other preservative which at any rate will prevent the specimen from going bad. If he wishes it mounted, he usually sends it to some laboratory where the work can be done for him. He will be glad to know of a simple method of preserving organs for colour.

It is important to have the specimen as fresh as possible; for this reason, objects removed at surgical operations keep their colour better than do those obtained at autopsies some hours or days after the patient died; for this very reason, if the colour of a specimen is to be preserved, there is no time to send it to a laboratory at any distance; the necessary solutions must be ready or made at home. The specimen must not be put into spirit first; the alcohol decolorises the blood very rapidly.

The first solution required is prepared as follows:

| | | | |
|---|---|---|---|
| Pure water ... ... | 5,000 c.c. | or | 8 pints |
| Pure formalin ... | 800 c.c. | ,, | 25 oz. |
| Potassium acetate... | 85 grams | ,, | 2½ oz. |
| Potassium nitrate ... | 45 grams | ,, | 1½ oz. |
| Glycerine ... ... | 200 c.c. | ,, | 6¼ oz. |

The specimen is immersed in this for twenty-four hours if it is small, or for forty-eight hours if it is large. The area of the tissue is of less importance than is its thickness. As a rule the part in which the colour preservation is most desired is at the surface, and care should be taken that the fluid can get at it freely. It is therefore a good thing to suspend the specimen in the liquid by a thread rather than to let it lie upon the bottom of the containing vessel. The fluid fixes the tissues, so that it is not easy to alter their shape afterwards; before immersing the organ, therefore, care should be taken to adjust it carefully to its natural shape—a stomach, for example, should be gently stuffed with cotton wool beforehand, and so on.

The next step is to take the specimen out of the fixing solution, and wash it in clean water; in the case of delicate structures this is best done by immersing them in a pail and gently moving them up and down in it. The specimen is then placed in the second solution, which is merely:—

Alcohol ... ... 80 per cent. to 90 per cent.

It is left in this for something like four to six hours; the alcohol no longer decolorises the blood that has been submitted to the first reagent.

The third solution is made up as follows:—

| | | | |
|---|---|---|---|
| Distilled water ... | 4,500 c.c. | or | 7 pints |
| Potassium acetate ... | 594 grams | or | 18½ oz. |

Dissolve the latter in the former; when cold, filter and add

Glycerine ... ... 1,500 c.c. or 2½ pints.

This is the final liquid, in which the specimen is to be mounted in the glass museum jar. The colour will remain indefinitely, but it is wise to keep the bottle in some place where it is not exposed to bright sunlight.

